# Modification of EBV Associated Lymphomagenesis and Its Immune Control by Co-Infections and Genetics in Humanized Mice

**DOI:** 10.3389/fimmu.2021.640918

**Published:** 2021-03-23

**Authors:** Patrick Schuhmachers, Christian Münz

**Affiliations:** Viral Immunobiology, Institute of Experimental Immunology, University of Zürich, Zürich, Switzerland

**Keywords:** cytotoxic lymphocytes, human immunodeficiency virus (HIV), Kaposi sarcoma associated herpesvirus (KSHV), HLA-DRB1*1501, mutant Epstein Barr viruses (EBVs)

## Abstract

Epstein Barr virus (EBV) is one of the most successful pathogens in humans with more than 95% of the human adult population persistently infected. EBV infects only humans and threatens these with its potent growth transforming ability that readily allows for immortalization of human B cells in culture. Accordingly, it is also found in around 1-2% of human tumors, primarily lymphomas and epithelial cell carcinomas. Fortunately, however, our immune system has learned to control this most transforming human tumor virus in most EBV carriers, and it requires modification of EBV associated lymphomagenesis and its immune control by either co-infections, such as malaria, Kaposi sarcoma associated herpesvirus (KSHV) and human immunodeficiency virus (HIV), or genetic predispositions for EBV positive tumors to emerge. Some of these can be modelled in humanized mice that, therefore, provide a valuable platform to test curative immunotherapies and prophylactic vaccines against these EBV associated pathologies.

## Introduction on EBV

The Epstein Barr virus (EBV) or human herpesvirus 4 (HHV4) is a ubiquitous human γ-herpesvirus that persistently infects more than 95% of the human population (1). In Sub-Saharan Africa, this percentage is already reached at 2 years of age, while in Europe and the US one third of the population acquires EBV at a later age (2). This delayed primary EBV infection bears the risk to develop into infectious mononucleosis (IM), an immunopathology due to massive anti-viral CD8^+^ T cell expansion and the accompanying cytokine release (3). Especially CD8^+^ T cells that recognize lytic EBV antigens, expressed during the viral infection program that produces viral particles, are increased to high frequencies during IM (4). Even so IM resolves in most cases, alterations in the resulting EBV specific immune response might be the reason for elevated risks for EBV associated Hodgkin’s lymphoma and multiple sclerosis (MS) (5, 6).

In addition to immunopathologies due to altered or increased immune responses to EBV infection, this virus is primarily known for its oncogenic potential (7). It was originally discovered in endemic Burkitt’s lymphoma (BL) of Sub-Saharan African children (8, 9). Furthermore, EBV can be found in a subset of Hodgkin’s lymphoma (HL), diffuse large B cell lymphoma (DLBCL) and immunoblastic lymphomas during immune suppression after transplantation, such as post-transplantation lymphoproliferative disease (PTLD), or during HIV co-infection (10). These are mostly B cell lymphomas, but also EBV associated natural killer (NK)/T cell lymphomas can occur, often after prolonged uncontrolled EBV infection (11). In addition, EBV is associated with epithelial cell derived nasopharyngeal carcinoma (NPC) and 10% of gastric carcinomas. These EBV associated malignancies mainly express latent EBV antigens that are not involved in infectious virus production but contain at least one of the main two EBV oncogenes, nuclear antigen 2 (EBNA2) and latent membrane protein 1 (LMP1) (12, 13). Latent EBV infection follows a pre-latent phase upon B cell entry during which a set of both lytic and latent antigens are expressed in order to support B cell activation, proliferation and survival [as reviewed in (14)]. During latent infection, which is the default gene expression program upon B cell infection by EBV, up to 6 EBNAs, two LMPs as well as non-translated Epstein–Barr virus-encoded small RNAs (EBERs) and miRNAs are expressed. This gene expression program is thought to drive EBV infected B cells after viral transmission *via* saliva in sub-mucosal secondary lymphoid tissues like tonsils into activation and differentiation to memory B cells, in which EBV then persists for life (15). From this memory B cell compartment, in which EBV only expresses non-translated RNAs, lytic reactivation and infectious virus production occurs after plasma cell differentiation, presumably after encountering the cognate antigen of the B cell receptor of the infected cell (16). At submucosal secondary lymphoid tissues this might lead to viral shedding into saliva for transmission. The distinct B cell differentiation stages and their respective EBV gene expression patterns can also be found in the EBV associated malignancies, and the respective B cell lymphomas (BL, HL and DLBCL) increase in frequency during iatrogenic or HIV induced immune suppression (10, 17). This suggests that immune responses prevent transition from premalignant latent EBV infections to overt tumors. Indeed, primary immunodeficiencies that affect individual genes map to cytotoxic lymphocytes and their ability to kill EBV infected B cells as the most important component of EBV specific immune control (18–20). In order to interrogate the function of the in patients identified genes, dissect the contribution of viral genes with EBV mutants and characterize the influence of co-infections *in vivo*, preclinical mouse models with reconstituted human immune cells (humanized mice) have been developed and their contribution to a better understanding of EBV infection, oncogenesis and immune control will be summarized in this review.

## EBV Infection, Immune Control, and Lymphomagenesis in Humanized Mice

To date humanized mice serve as a reliable model to study pathogens that exclusively target humans. In the past decades, several humanized mouse models were established that responded to infection with EBV and allowed for assessing the importance of host immune factors as well as viral proteins during an infection. In this review, we will primarily focus on NOD/Shi-scid/IL-2Rγ^null^ (NOG) and NOD/LtSz-scid IL2Rγ^null^ (NSG) mice with and without HLA-A2 transgenes as well as NSG or NOD/LtSz-scid mice implanted with human fetal liver and thymus tissue (BLT) that were all either neonatally or as adult mice reconstituted with human immune system components by transfer of human CD34^+^ hematopoietic progenitor cells (HPCs) or of cord blood often after CD34^+^ HPC depletion. Most studies with EBV infection have been performed in these particular humanized mouse models and these were consistently permissive for multiple EBV strains (21–25), mirroring acute infection as well as EBV associated lymphomagenesis of humans. A more complete overview of humanized mouse models was recently published (26). As is the case for humans, human B cells constitute the main reservoir for EBV in humanized mice, enabling viral replication and lymphoproliferation. EBV infection of humanized mice was therefore marked by viral loads in blood and secondary lymphoid organs (22, 24, 27, 28).

Analogously to acute symptomatic primary infection in humans (29–31), the number of NK cells in peripheral blood and spleen of humanized NSG mice increases starting at three weeks of EBV infection and peaks at week four. The NK cell response constitutes an important measure to prevent uncontrolled lytic EBV infection and to bridge the time until adaptive T cell responses are primed. Indicative of this is that depletion of NK populations in humanized NSG mice resulted in higher viral loads and tumor incidence (32, 33).

Initial control by NK cells is succeeded by priming and expansion of cytotoxic CD8^+^ T lymphocytes (CTLs) in peripheral blood. Those CTLs are mainly specific for lytic antigens (22, 25, 34, 35) and exhibit a cytolytic effector profile determined by high expression of activation molecules like HLA-DR or 2B4 and cytotoxic effector molecules such as Granzyme B (22, 25, 35). Consequently, expression of HLA-DR positively correlates with increasing viral loads in infected humanized NSG mice (36). In contrast to CTL expansion, CD4^+^ helper T cells do not expand to a similar degree which is why an inversion of the CD8 to CD4 T cell ratio is one of the hallmarks of EBV infection in humanized mice as is in humans suffering from IM (25, 34). Despite lower expansion rates and total numbers, CD4^+^ T cell help seems to be required to tackle the infection in humanized mice since CD4^+^ T cell depletion prior to infection results in higher viral loads (22). This corresponds to human data depicting a cytolytic effector function of EBV specific CD4^+^ T cells during infection (37, 38).

In contrast to T cell responses, antibody mediated responses to EBV are not yet as well characterized in humanized mice. In humanized NOG and NSG mice, IgM responses to BFRF3 and EBNA1, respectively, were observed. Detection of EBV specific IgG antibodies, however, has proven to be more difficult. This drawback might arise from deficiencies in germinal center formation and therefore difficulties in antibody isotype class switching as well as inefficient B cell development in several humanized mouse systems (22, 39–43). There are, however, promising developments in the generation of humanized mice capable of mounting IgG responses to pathogens (44, 45).

Despite active immune control of EBV infection, lymphoma formation can be observed in humanized mice. The degree of lymphomagenesis is thereby dependent on the amount of viral particles with which the animal is challenged. Humanized NSG mice, for example, present in 20 – 30% of cases with disseminated lymphomas in spleen, liver, lymph nodes or kidney when challenged intraperitoneally with high dose EBV (10^5^ infectious particles) for four to five weeks (46, 47). Other mouse models, as for example humanized BALB/c Rag2^null^IL2rγ^null^ Sirpa^NOD^ (BRGS) mice, present with higher lymphoma incidences of up to 75% while surviving four weeks of infection (48). Besides that, lymphomagenesis may be dependent on genomic or host immunologic alterations which will be discussed in the following sections.

## Altered EBV Pathology Due to Genetic Alterations in the Viral Genome

Since the discovery that humanized mice are susceptible to EBV infection and allow for the identification of host immune factors in response to the pathogen, several groups started assessing the importance of viral genes during infection. For example, infection of a humanized BRGS mouse with type 1 or 2 EBV strains was examined. The two EBV strains differ mainly in their genetic sequence of the latent genes EBNA2, EBNA3A and EBNA3C (49–51) and, consequently, in their ability to transform infected cells *in vitro*. More importantly, in contrast to EBV type 1, EBV type 2 was additionally observed to be human T cell tropic which could explain findings of EBV related human T cell lymphomas (48). *Coleman and colleagues* were able to reproduce T cell tropism of EBV type 2 *in vivo*. However, the infection of humanized mice with either strain resulted in comparable degrees of viral replication and lymphomagenesis contrasting the *in vitro* findings. Lymphomas caused by both strains exhibited similar features, resembling diffuse large B cell lymphomas (DLBCL) and expressing all latent EBV gene products (48, 52). The strain specific differences that underlie *in vivo* infection seem, for now, to be limited to lower LMP1 expression levels and higher lytic activity in EBV type 2 infected animals (52). Still, the reason why the two EBV strains developed different strategies, remains elusive. In addition to EBV type 2 that is primarily found in Sub-Saharan Africa (53), also Asian EBV strains present with higher lytic EBV replication, and this also extends to infections of humanized mice (21, 54, 55).

**Figure 1:**
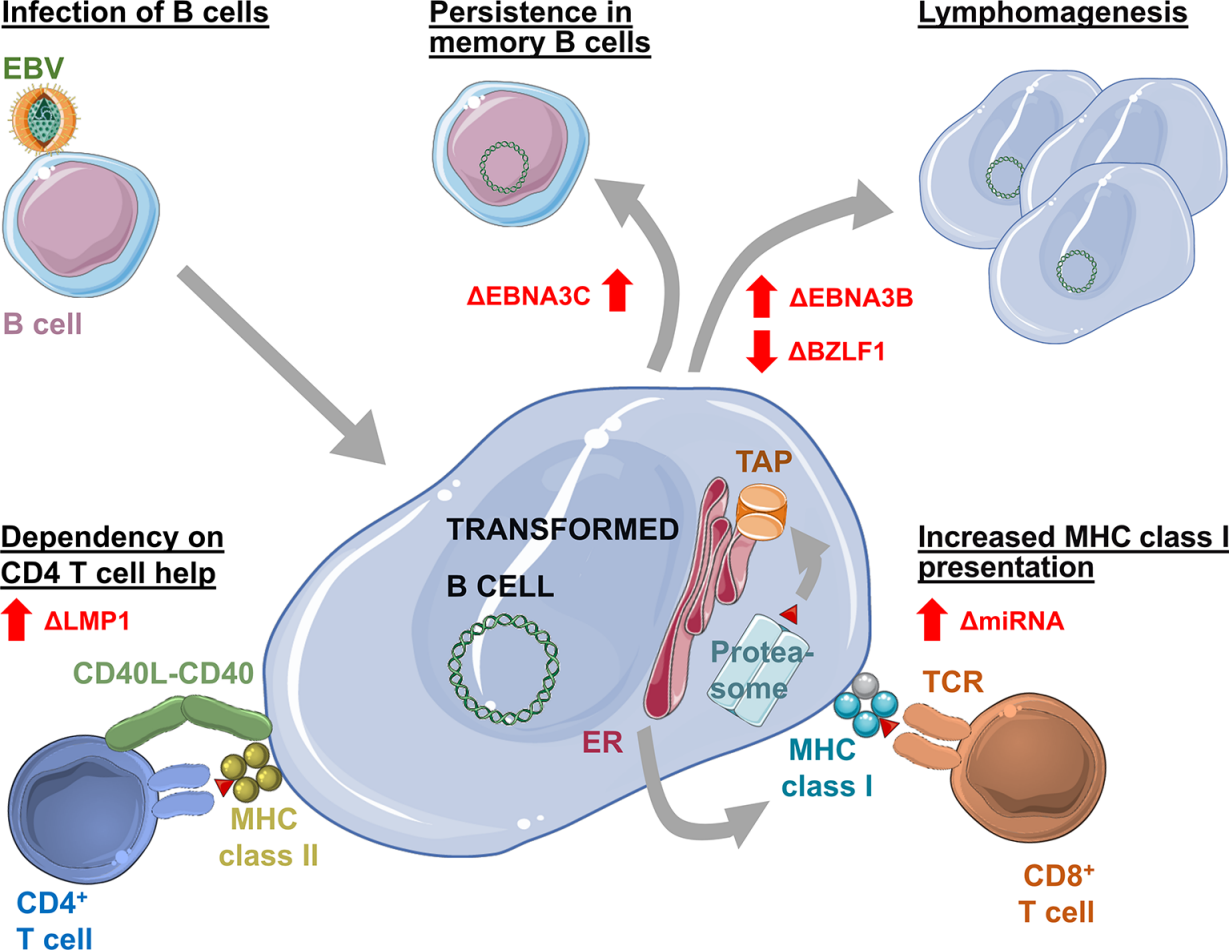
Changes in Epstein Barr virus (EBV) infection, pathogenesis and immune control as revealed by infection of humanized mice with mutant viruses. Elimination of EBNA3C (ΔEBNA3C) allows establishment of persistent EBV infection without transformation. Loss of EBNA3B (ΔEBNA3B) increases and loss of BZLF1 (ΔBZLF1) decreases EBV associated lymphomagenesis. Loss of viral miRNAs (ΔmiRNA) leads to increased expression of the antigen processing machinery for MHC class I presentation, allowing for improved immune control by CD8^+^ T cells. Deficiency in LMP1 (ΔLMP1) causes dependency on CD4^+^ T cell help for EBV induced B cell transformation.

Apart from defining differences between the virus strains, various gene loci of EBV type 1 were extensively studied in the past decades using humanized mice (**Figure 1**). *White and colleagues* investigated the contribution of EBNA3B to infection. In their study, they infected humanized NSG mice with the B95-8 (EBV type 1) strain of EBV which lacked the EBNA3B gene locus. Interestingly, the absence of EBNA3B led to higher tumor incidences in those mice which *White and colleagues* assigned to higher replicative activity of infected cells and a lower level of T cell infiltration into tumors due to decreased expression of chemo-attractants as for example CXCL9 and CXCL10 (56). A lower degree of T cell infiltration is thereby in line with EBNA3B being often targeted by T cells (57).

In addition to T cell infiltration, the importance of MHC class I restricted antigen presentation for EBV specific immune control by CD8^+^ T cells in humanized mice was demonstrated with a B95-8 virus that lacks the viral miRNAs (36). These compromise antigen processing for MHC class I presentation (58). In their absence EBV is more efficiently immune controlled in humanized mice in a CD8^+^ T cell dependent manner. In contrast to miRNA deficiency, EBER knock-out EBV infects humanized mice similarly to wild-type virus (59), except for the increased inflammation promoting potential of EBER2 of some Asian EBV strains (55).

Other studies on B95-8 viruses lacking the EBNA3A or EBNA3C gene locus highlighted that these genes are dispensable for establishment of persistent infection *in vivo*. Despite findings suggesting that EBNA3A or 3C knockout viruses are hardly able to transform B cells *in vitro*, persistence was established in secondary lymphoid organs and blood over a period of three months and T cell responses were mounted against the virus. Lack of EBNA3A or 3C, however, seems to result in decreased aggressiveness of EBV infection as viral loads were lower and the tumorigenic potential was presumably lost (60). Studies in cord blood reconstituted humanized NSG mice in which T cells might exert less immune control due to efficient inhibitory receptor engagement and in which higher frequencies of EBV associated lymphomas are observed (61), came to similar conclusions regarding a decreased aggressiveness in the absence of EBNA3A and 3C. However, EBNA3A mutants could not only establish persistent infection in this model, but tumorigenic potential was delayed and not lost. Still, both studies observed decreased LMP1 expression levels in infected cells in spleen and tumor tissue (60, 62). In addition, the use of a complete EBNA3A knockout virus (60) compared to an EBV mutant with only decreased EBNA3A expression (62) might explain differences in the viruses’ tumorigenic potential. In the same cord blood reconstituted humanized NSG mouse model, EBNA3C knockout EBV was able to cause lymphomas with lower frequency. Similar to EBNA3A hypomorphic EBV, lymphoma formation seemed to be delayed (52). Therefore, in humanized mice with diminished immune control, decreased EBNA3A or absent EBNA3C expression might still allow for the delayed development of EBV associated diffuse large B cell lymphomas.

Furthermore, LMP1 and 2 might also be dispensable for EBV infection in humanized mice (63, 64), but their absence delays lymphomagenesis. In the case of LMP1 deficiency the observed lymphoma formation required CD4^+^ T cell help (63).

Surprisingly also lytic EBV reactivation, at least early lytic gene expression, seems to promote lymphomagenesis in humanized mice. B95-8 EBV infection caused less tumors in the absence of the immediate early transactivation factor BZLF1 that induces lytic reactivation (23, 46). Vice versa, BZLF1 promotors that enhance lytic EBV infection are associated with increased lymphoma formation (65, 66). Infection of humanized mice with mutant EBV viruses can therefore reveal the function of genetic variability or viral gene products, enabling further insights into the life cycle of EBV *in vivo*.

## Modification of EBV Specific Immune Control by Host Genetics and Manipulation of Human Immune Compartments

In addition to testing mutant EBV viruses and different viral isolates *in vivo*, humanized mice also allow interrogation of human genetic variation, gene products and leucocyte compartments during EBV infection, oncogenesis and immune control. With regards to genetic variation IM and elevated antibody responses against EBV nuclear antigen 1 (EBNA1) have been found to synergize with the MHC class II molecule HLA-DRB1*1501 to increase risk for the development of MS (5). Indeed, HLA-DRB1*1501 restricted EBNA1 specific CD4^+^ T cell responses are also elevated in MS patients (67–69). However, despite elevated T cell responses to EBV infection in humanized mice that have been reconstituted from HLA-DRB1*1501 donors, these animals experience higher viral loads (70). Furthermore, the respective HLA-DRB1*1501 restricted CD4^+^ T cells that recognize EBV transformed B cells (lymphoblastoid cell lines or LCL) cross-react with myelin basic protein (MBP), an autoantigen in MS. These findings suggest that EBV is inefficiently immune controlled in the context of HLA-DRB1*1501 and that the resulting increased numbers of EBV infected B cells might stimulate in turn myelin antigen specific autoreactive T cell responses to cause MS.

Indeed CD4^+^ T cell responses seem essential to maintain efficient immune control of EBV in humanized mice. Both, iatrogenic immune suppression with tacrolimus (FK506) that mainly affects CD4^+^ T cell activation and expansion after EBV infection of humanized mice, and CD4^+^ T cell depletion by HIV co-infection leads to elevated viral loads and increased EBV associated B cell lymphoma formation (71, 72). During HIV co-infection CD8^+^ T cell depletion does not further increase EBV viral loads or lymphoma formation (72). This suggests that HIV induced CD4^+^ T cell depletion compromises T cell help to maintain protective CD8^+^ T cell function because CD8^+^ T cell depletion during only EBV infection of humanized mice significantly affects immune control (22, 36, 72, 73). Moreover, antibody mediated depletion of both CD4^+^ and CD8^+^ T cells increases viral loads and associated tumors in EBV infected humanized mice (22, 43). In addition, antibody blocking of 2B4, a co-stimulatory molecule on cytotoxic lymphocytes that uses SLAM-associated protein (SAP), and SAP is mutated in X-linked lymphoproliferative disease 1 (XLP1) thereby predisposing for EBV associated pathology, increases EBV viral loads and lymphomagenesis in humanized mice (73). 2B4 blocking on top of CD8^+^ T cell depletion does not lower EBV specific immune control further, suggesting that 2B4 is mainly required on CD8^+^ T cells to suppress EBV infection. SAP deficiency is, however, also associated with loss of invariant NKT cells (74) which have been shown to restrict EBV transformed B cells in humanized mice (75, 76). This could also contribute to compromised immune control of EBV in XLP1. Moreover, antibody blocking of PD-1, an inhibitory receptor on both effector and regulatory T cells, increases EBV loads and lymphomagenesis in infected humanized NSG mice engrafted with human CD34^+^ HPCs (26, 35). This loss of immune control correlates with immune suppressive cytokine and regulatory T cell amounts in anti-PD-1 treated and EBV infected humanized mice. Strikingly, blocking PD-1 and/or CTLA-4 in cord blood reconstituted humanized NSG mice resulted in decreased tumor formation (61). As cord blood reconstituted humanized NSG mice seem to develop weaker T cell mediated immune control after EBV infection and allow for higher frequencies of EBV associated lymphomas, especially after infection with the lytic M81 EBV strain, PD-1 and CTLA-4 blockade might be required to strengthen this immune control (61) which seems to be in part based on Vγ9Vδ2 T cells (77). Thus, composition of the T cell compartment and balance between regulatory and anti-viral T cells might determine the outcome of PD-1 and CTLA-4 blockade. However, genetic loss of CTLA-4 seems to be associated with EBV associate pathologies in some of the affected individuals (78). Similarly, PD-1 blockade seems to cause loss of EBV specific immune control and brain homing of EBV infected B cells in a subgroup of treated patients (79).

In addition to T cells, innate lymphocytes contribute to EBV specific immune control. Protection against EBV infection of humanized mice has been shown for NK, NKT and Vγ9Vδ2 T cells (32, 33, 75, 77, 80). Early differentiated NKG2A^+^KIR^-^ NK cells restrict primarily lytic EBV replication and degranulate their cytotoxic machinery toward lytically EBV replicating BL cells (29, 32). In contrast, depletion of plasmacytoid dendritic cells, the main hematopoietic source of type I interferon (IFN) during viral infections, does not significantly influence EBV infection of humanized mice (81). Accordingly, type I IFN signaling deficiencies do not predispose for EBV associated pathologies (19). These studies demonstrate that humanized mice can be used to interrogate the role of genetic variations, of leucocyte compartments and of their receptors in EBV specific immune control.

## Alteration of EBV Associated Pathogenesis by Co-Infection

**Figure 2:**
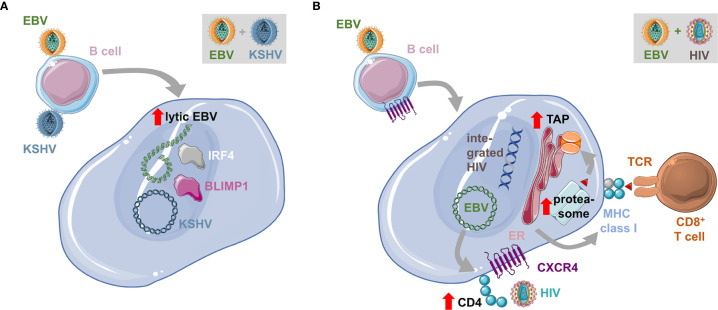
Influence of co-infections on EBV transformed B cells. **(A)** Co-infection with the Kaposi sarcoma associated herpesvirus (KSHV) transactivates lytic EBV replication and is associated with plasma cell differentiation, characterized for example by BLIMP1 and IRF4 expression. This leads to enhanced lymphomagenesis and the resulting tumors have characteristics of primary effusion lymphomas (PELs). **(B)** EBV infection of B cells up-regulates CD4 and with the sustained expression of CXCR4 renders EBV transformed B cells susceptible to infection with the human immunodeficiency virus (HIV). This leads to the integration of the reverse transcribed HIV genome into the host cell genome. Double-infected B cells up-regulate the antigen processing machinery for MHC class I presentation and are therefore efficiently immune controlled by CD8^+^ T cells.

In addition to HIV infection, some EBV associated malignancies are also observed during additional co-infections. Endemic BL can be primarily observed in geographic areas of holoendemic exposure to the malaria parasite *Plasmodium falciparum* (82, 83). In Sub-Saharan Africa, where Denis Burkitt described this tumor for the first time (84), Kaposi sarcoma associated herpesvirus (KSHV) or human herpesvirus 8 (HHV8) a another pathogen with which EBV interacts during co-infections is also highly prevalent (85). Both EBV and KSHV are found in the tumor cells of 90% of primary effusion lymphomas (PELs) (10). Moreover, KSHV infection seems to benefit from EBV co-infection for persistence (47, 86–88). Humanized mice that are infected with both KSHV and EBV develop B cell lymphomas with higher incidence (47). The developing lymphomas harbor both EBV and KSHV (**Figure 2**). They present with hallmarks of plasma cell differentiation that is characteristic for PELs (89). Interestingly, this plasma cell differentiation that is also in healthy EBV carriers associated with lytic EBV replication (16), causes elevated induction of at least early lytic EBV reactivation in PEL-like tumors of double-infected humanized mice (47). Co-infection with an EBV mutant that can no longer activate lytic infection (BZLF1 deficient EBV), reduces lymphomagenesis during EBV and KSHV co-infection of humanized mice, suggesting that the transactivated lytic EBV gene expression might contribute to conditioning of the tumor microenvironment for efficient growth (7).

HIV co-infection also does more than just suppress EBV specific immune control. It was noted that anti-retroviral therapy (ART) reduced immunoblastic EBV associated lymphomas in HIV infected individuals, while BL and HL frequencies did not decline (17). Similarly, in humanized mice HIV co-infection influences EBV infected B cells directly (72). It was found that HIV is capable to infect EBV transformed B cells due to CD4 up-regulation during EBV infection and maintained CXCR4 expression on human B cells (**Figure 2**). HIV also integrates and replicates in EBV infected B cells but alters their gene expression pattern for enhanced antigen processing toward MHC class I restricted antigen presentation. Accordingly, HIV and EBV infected B cells are efficiently recognized by both EBV and HIV specific CD8^+^ T cells and mainly accumulate in double-infected humanized mice after CD8^+^ T cell depletion. Future studies will need to show if double-infected B cells alter EBV associated lymphoma formation over a longer observation period, independent of HIV induced immune suppression.

## Conclusions and Outlook

In summary, the development of humanized mice revolutionized the study of pathogens exclusive to humans such as EBV. The possibility to use small rodents instead of endangered New World monkeys (90, 91) greatly facilitated the research on host immune factors and viral genes during infection. Various humanized mouse models are consistently permissive for infection with and allow replication of EBV. Even more promising, these models mimic human lymphoproliferative diseases and the reaction of human immune system components to infection. In addition, host and virus genetic alterations and their implications in disease outcome may be more extensively studied in humanized mice which may not be comparably easy in humans. Further studies on host susceptibility factors like HLA-DRB1*1501 are needed to assess additional groups at risk of developing EBV associated diseases and to develop personalized treatments in the future. Similarly, the interplay between EBV and other co-infecting pathogens is difficult to assess in humans but may be elucidated in humanized mice. Thus, with humanized mice it became feasible to study not only contributions of single immune cell types or single molecules to infection outcome but also to investigate the importance of virus and host genetics. The findings resulting from infection models in humanized mice thereby enable applied research on EBV vaccines, or the development of new treatments against EBV induced lymphomas.

Despite these advantages there are still limitations to each of the humanized mouse models employed. The biggest limitation with respect to EBV infection is that humanized mice do not support infection of oropharyngeal epithelia. In humans the infection starts in the oropharynx with a complex interplay between submucosal B cells and polarized epithelia. Therefore, EBV’s complete life cycle cannot be mimicked thus far. Furthermore, most models failed to mount proper antibody responses to EBV which is why their contribution to infection could not yet be well studied using a mouse model. Reports on new humanized mouse models with improved seroconversion to IgG, however, seem promising in solving this problem (44, 92). A further limitation of the presented EBV infection models is that in most cases lymphomas with expression of all latent EBV genes develop. As a result, the in humans more prominent HL or BL with restricted latent gene expression cannot currently be modelled in humanized mice and therefore such models still have to be developed. Along these lines infection with EBNA2 deficient EBV has recently been reported to cause lymphomas with some HL characteristics and might be further explored to gain insights into this EBV associated malignancy (93). With further developments of humanized mice in the upcoming years we may be able to reveal even minor host and viral genetic as well as host immune factors that contribute to control of EBV infection. Thus, we may be able to use the gained knowledge to design vaccines or therapies against this most transforming human tumor virus.

## Author Contributions

PS and CM wrote the manuscript. All authors contributed to the article and approved the submitted version.

## Funding

Our research is supported by Cancer Research Switzerland (KFS-4962-02-2020), CRPP-Precision^MS^ and HMZ ImmunoTargET of the University of Zurich, the Cancer Research Center Zurich, the Baugarten Foundation, the Sobek Foundation, the Swiss Vaccine Research Institute, Roche, Novartis, Novartis Foundation (20B099), and the Swiss National Science Foundation (310030B_182827, 310030L_197952/1 and CRSII5_180323).

## Conflict of Interest

The authors declare that the research was conducted in the absence of any commercial or financial relationships that could be construed as a potential conflict of interest.
